# Supplementary material for: High expression of PIMREG predicts poor survival outcomes and is correlated with immune infiltrates in lung adenocarcinoma
Source: PeerJ. 2021 Jul 6;9:e11697. doi: 10.7717/peerj.11697 (PMC8269662; doi:10.7717/peerj.11697)
Supplement: Supplemental Information 1 [file peerj-09-11697-s001.doc]

Supplementary Table 1. Univariate analysis and multivariate analysis of the correlation between clinicopathological characteristics and DSS in LUAD

| Characteristics | Total(N) | HR(95% CI) Univariate analysis | P value Univariate analysis | HR(95% CI) Multivariate analysis | P value Multivariate analysis | | |
| --- | --- | --- | --- | --- | --- | --- | --- |
| T stage (T2&T3&T4 vs. T1) | 466 | 1.747(1.125-2.714) | 0.013 | 1.172(0.587-2.338) | 0.653 |  |  |
| N stage (N1&N2&N3 vs. N0) | 457 | 2.795(1.919-4.071) | <0.001 | 2.269(0.821-6.272) | 0.114 |  |  |
| M stage (M1 vs. M0) | 327 | 2.480(1.278-4.811) | 0.007 | 2.176(0.781-6.062) | 0.137 |  |  |
| Pathologic stage (Stage II&Stage III&Stage IV vs. Stage I) | 461 | 3.519(2.350-5.271) | <0.001 | 0.824(0.283-2.395) | 0.722 |  |  |
| Primary therapy outcome (PD&SD&PR vs. CR) | 408 | 4.284(2.883-6.366) | <0.001 | 5.182(2.900-9.260) | <0.001 |  |  |
| Residual tumor (R1&R2 vs. R0) | 320 | 5.020(2.479-10.167) | <0.001 | 6.177(2.077-18.371) | 0.001 |  |  |
| Gender (Male vs. Female) | 469 | 0.956(0.658-1.390) | 0.815 |  |  |  |  |
| Age (>65 vs. <=65) | 459 | 1.039(0.713-1.513) | 0.842 |  |  |  |  |
| Race (White vs. Asian&Black or African American) | 423 | 1.089(0.630-1.883) | 0.759 |  |  |  |  |
| Anatomic neoplasm subdivision (Right vs. Left) | 456 | 1.109(0.749-1.641) | 0.606 |  |  |  |  |
| Anatomic neoplasm subdivision2 (Peripheral Lung vs. Central Lung) | 167 | 1.207(0.630-2.312) | 0.57 |  |  |  |  |
| number pack years smoked (>=40 vs. <40) | 318 | 0.904(0.569-1.437) | 0.67 |  |  |  |  |
| Smoker (Yes vs. No) | 455 | 1.013(0.585-1.755) | 0.962 |  |  |  |  |
| Tumor status (With tumor vs. Tumor free) | 450 | 1487458274.795(0.000-Inf) | 0.993 |  |  |  |  |
| TP53 status (Mut vs. WT) | 465 | 1.335(0.920-1.937) | 0.128 |  |  |  |  |
| KRAS status (Mut vs. WT) | 465 | 0.842(0.536-1.324) | 0.457 |  |  |  |  |
| PIMREG (High vs. Low) | 469 | 1.868(1.276-2.735) | 0.001 | 1.536(0.851-2.771) | 0.154 |  |  |
