# Supplementary material for: High expression of PIMREG predicts poor survival outcomes and is correlated with immune infiltrates in lung adenocarcinoma
Source: PeerJ. 2021 Jul 6;9:e11697. doi: 10.7717/peerj.11697 (PMC8269662; doi:10.7717/peerj.11697)
Supplement: Supplemental Information 2 [file peerj-09-11697-s002.doc]

Supplementary Table 2. Univariate analysis and multivariate analysis of the correlation between clinicopathological characteristics and PFI in LUAD

| Characteristics | Total(N) | HR(95% CI) Univariate analysis | P value Univariate analysis | HR(95% CI) Multivariate analysis | P value Multivariate analysis | | |
| --- | --- | --- | --- | --- | --- | --- | --- |
| T stage (T2&T3&T4 vs. T1) | 501 | 1.819(1.319-2.510) | <0.001 | 1.379(0.819-2.321) | 0.227 |  |  |
| N stage (N1&N2&N3 vs. N0) | 492 | 1.667(1.261-2.205) | <0.001 | 1.010(0.511-1.994) | 0.977 |  |  |
| M stage (M1 vs. M0) | 360 | 1.620(0.913-2.876) | 0.099 | 0.790(0.320-1.952) | 0.61 |  |  |
| Pathologic stage (Stage II&Stage III&Stage IV vs. Stage I) | 496 | 2.169(1.641-2.868) | <0.001 | 0.908(0.439-1.878) | 0.794 |  |  |
| Primary therapy outcome (PD&SD&PR vs. CR) | 419 | 3.793(2.820-5.104) | <0.001 | 2.700(1.659-4.395) | <0.001 |  |  |
| Residual tumor (R1&R2 vs. R0) | 352 | 3.303(1.771-6.160) | <0.001 | 3.264(1.336-7.972) | 0.009 |  |  |
| Gender (Male vs. Female) | 504 | 1.072(0.815-1.411) | 0.617 |  |  |  |  |
| Age (>65 vs. <=65) | 494 | 1.106(0.837-1.460) | 0.479 |  |  |  |  |
| Race (White vs. Asian&Black or African American) | 446 | 1.093(0.722-1.655) | 0.673 |  |  |  |  |
| Anatomic neoplasm subdivision (Right vs. Left) | 490 | 1.067(0.801-1.421) | 0.658 |  |  |  |  |
| Anatomic neoplasm subdivision2 (Peripheral Lung vs. Central Lung) | 182 | 1.073(0.673-1.713) | 0.766 |  |  |  |  |
| number pack years smoked (>=40 vs. <40) | 345 | 1.011(0.723-1.414) | 0.95 |  |  |  |  |
| Smoker (Yes vs. No) | 490 | 0.946(0.637-1.404) | 0.782 |  |  |  |  |
| Tumor status (With tumor vs. Tumor free) | 450 | 12.918(8.871-18.812) | <0.001 | 6.297(3.755-10.558) | <0.001 |  |  |
| TP53 status (Mut vs. WT) | 499 | 1.048(0.796-1.381) | 0.737 |  |  |  |  |
| KRAS status (Mut vs. WT) | 499 | 0.998(0.723-1.376) | 0.988 |  |  |  |  |
| PIMREG (High vs. Low) | 504 | 1.583(1.201-2.086) | 0.001 | 1.725(1.100-2.703) | 0.017 |  |  |
