# Supplementary figures and images for: High expression of PIMREG predicts poor survival outcomes and is correlated with immune infiltrates in lung adenocarcinoma
Source: PeerJ. 2021 Jul 6;9:e11697. doi: 10.7717/peerj.11697 (PMC8269662; doi:10.7717/peerj.11697)

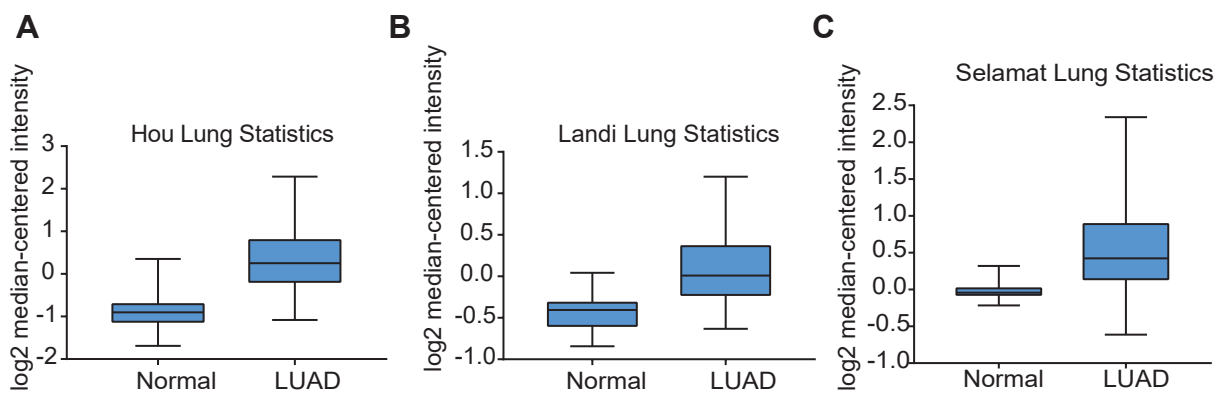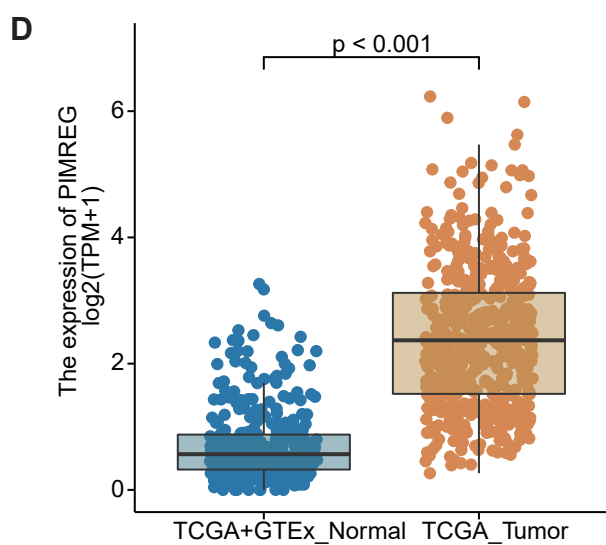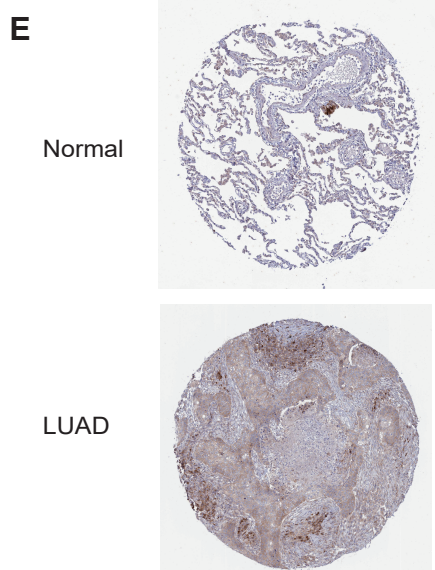

Supplement: Supplemental Information 5 — (A–C) PIMREG mRNA expression in normal lung tissues and LUAD tissues was detected using the Oncomine database. (D) PIMREG mRNA expression in normal lung tissues and paired LUAD tissues was analyzed based on TCGA and GTEx datasets. (E) HPA database were applied to analyze the characterization of PIMREG expression. [file peerj-09-11697-s005.pdf]
